# Hydrogen Peroxide-Releasing Hydrogel-Mediated Cellular Senescence Model for Aging Research

**DOI:** 10.34133/bmr.0161

**Published:** 2025-03-14

**Authors:** Shibo Wei, Phuong Le Thi, Yan Zhang, Moon-young Park, Khanh Do, Thi Thai Thanh Hoang, Nyssa Morgan, Tam Dao, Jimin Heo, Yunju Jo, You Jung Kang, Hansang Cho, Chang-Myung Oh, Young C. Jang, Ki-Dong Park, Dongryeol Ryu

**Affiliations:** ^1^ Department of Biomedical Science and Engineering, Gwangju Institute of Science and Technology, Gwangju 61005, Republic of Korea.; ^2^ Institute of Applied Materials Science, Vietnam Academy of Science and Technology, Ho Chi Minh City 700000, Vietnam.; ^3^ Department of Intelligent Precision Healthcare Convergence, Sungkyunkwan University School of Medicine, Suwon 16419, Republic of Korea.; ^4^ Department of Orthopaedics, Emory Musculoskeletal Institute, Emory University School of Medicine, Atlanta, GA, USA.; ^5^ Wallace H. Coulter Department of Biomedical Engineering, Georgia Institute of Technology and Emory University School of Medicine, Atlanta, GA 30332, USA.; ^6^ Atlanta VA Medical Center, Decatur, GA, USA.; ^7^ Parker H. Petit Institute for Bioengineering and Biosciences, School of Biological Sciences, Georgia Institute of Technology, Atlanta, GA 30332, USA.; ^8^ Department of Medicinal Chemistry and Pharmacology, University of Science and Technology, Daejeon 34113, Republic of Korea.; ^9^ Department of Molecular Science and Technology, Ajou University, Suwon 16499, Republic of Korea.

## Abstract

Cellular senescence, a process that induces irreversible cell cycle arrest in response to diverse stressors, is a primary contributor to aging and age-related diseases. Currently, exposure to hydrogen peroxide is a widely used technique for establishing in vitro cellular senescence models; however, this traditional method is inconsistent, laborious, and ineffective in vivo. To overcome these limitations, we have developed a hydrogen peroxide-releasing hydrogel that can readily and controllably induce senescence in conventional 2-dimensional cell cultures as well as advanced 3-dimensional microphysiological systems. Notably, we have established 2 platforms using our hydrogen peroxide-releasing hydrogel for investigating senolytics, which is a promising innovation in anti-geronic therapy. Conclusively, our advanced model presents a highly promising tool that offers a simple, versatile, convenient, effective, and highly adaptable technique for inducing cellular senescence. This innovation not only lays a crucial foundation for future research on aging but also markedly accelerates the development of novel therapeutic strategies targeting age-related diseases.

## Introduction

As we age, our body’s ability to cope with stress progressively deteriorates. As a consequence, cellular degenerations such as DNA damage, telomere attrition, and oxidative and nitrosative stress accumulates as function of time [1,2]. Under these aging-associated stressors, somatic and stem cells can irreversibly halt proliferation and transition to senescent state in many tissues [3]. Cellular senescence has emerged as a significant contributor in tissue aging as well as a potential therapeutic target for a variety of age-related diseases [4]. According to statistics from the United Nations in 2015, the number of elderly people (aged 60 years or above) is dramatically increasing and is estimated to nearly double from 12% to 22% of the population by 2050 [5]. As a result, it is pivotal to enhance our understanding of the underlying mechanisms of aging and discover translational geroscience approaches to mitigate age-dependent pathologies in the future. Therefore, establishing a reliable cellular senescence model that recapitulates in vivo human aging and age-related diseases is crucial and a necessity for geroscience research.

The basic premise of the oxidative stress theory of aging is that an increase in production of reactive oxygen species overwhelms the antioxidant defense system leading to oxidative modification of key macromolecules such as protein, lipids, and DNA. These oxidative stress conditions and deleterious changes in redox signaling are thought to be the one of the main causes of aging and age-related diseases. The oxidative stress theory of aging has been extensively tested in different animal as well as in vitro cell models. Due to its causative role in aging, several approaches have been employed to promote senescence by inducing oxidative stress, such as exposure to ethanol, tert-butyl hydroperoxide, ultraviolet light, and hydrogen peroxide (H_2_O_2_)—a natural source of oxidative stress and redox regulator in vivo and in vitro [6]. However, these traditional techniques yielded inconsistent, laborious, and, for the most part, failed to capture intricate physiological and pathological changes of cellular senescence in vivo [7]. In this regard, it is imperative to develop advanced strategies of replicating senescence in the context of aging research.

Traditional 2-dimensional (2D) cell cultures using cells from genetic or chronologically aged animal models have been widely utilized as tools to enhance our understanding of age-dependent disorders in vitro [8]. However, there are several constraints and limitations in these approaches, such as their inaccurate simulations of intra-organ interactions and physiological manifestations in living tissues [9]. Moreover, comparable “one-size-fits-all” findings drawn from conventional models have not always provided sufficient evidence for clinical treatments. As a result, over the last decade, marked advances have been made in integrating stem cell techniques to simulate in vivo physiology more accurately; there must be a rapid expansion in relevant in vitro model development. Additionally, there is recent emphasis on the importance of personalized medicine, allowing for precise drug prescription, disease process preemption, and a shift from reaction to prevention, which is especially relevant in aged patients and anti-aging therapy, where comorbidities are the norm [10]. In this context, precise patient-derived disease models are emerging in urgent demand. Scientists have developed 3-dimensional (3D) organ models, like organ-on-chips (OOCs) and induced pluripotent stem cell (iPSC)-derived organoids, which imitate disease models with remarkable fidelity. As human “avatars”, OOCs help establish patient-specific phenotypic models of rare diseases [11]. Organoids are miniaturized and self-organized replicas of organs produced with accurate micro-anatomy [12]. Because these 2 advanced strategies aim to reproduce the cellular niche of organs and their intrinsic properties, they are employed to investigate developmental organogenesis, disease progression, and homeostasis. However, one of the notable limitations of iPSC-derived organs are how restrictive they can be when researching the effects of these human “avatars” on aging. DNA methylation patterns, commonly known as methylation clocks, are closely associated with aging. The methylation clock is composed of processes of important epigenetic modification and is a key component in normal differentiation and age-related disease development [13]. Although iPSC-derived organs are functionally mature, they do not retain age-related transcriptomic or epigenomic profiles [14]. In other words, when we expand iPSCs, or even fibroblasts (source of iPSCs) isolated from the elderly into these human “avatars”, they still may not realistically represent age progression, because their methylated signatures are lost through passaging [15]. To overcome this, it is requisite to induce cellular senescence into OOC and organoids for aging reproduction. Nevertheless, existing inadequacies in traditional procedures mean that there is currently no practically feasible tool for developing aging models in 3D culture.

Furthermore, it is a key determinant to aging and age-related diseases that senescent cells accumulate in different tissues, leading to inevitable and progressive loss of the tissues’ ability to recover from stress. Recently, research on the treatment of senescent-associated disorders has prompted interest in senolytics, a class of drugs that can selectively eliminate senescent cells [16,17]. Evidence has shown that cellular senescence is a causal mechanism in the generation of age-related phenotypes. Alternatively, senescent cell elimination using senolytics can prevent or postpone tissue malfunction and extend health span [18,19]. Scientists have discovered several potential anti-aging compounds, including dasatinib [20,21], quercetin [22,23], fisetin [24], navitoclax [25], piperlongumine [26], azithromycin, and roxithromycin [27]. However, there is still a lack of simple and effective in vitro models for screening senolytics or assessing their efficacy.

**Fig. 1.**
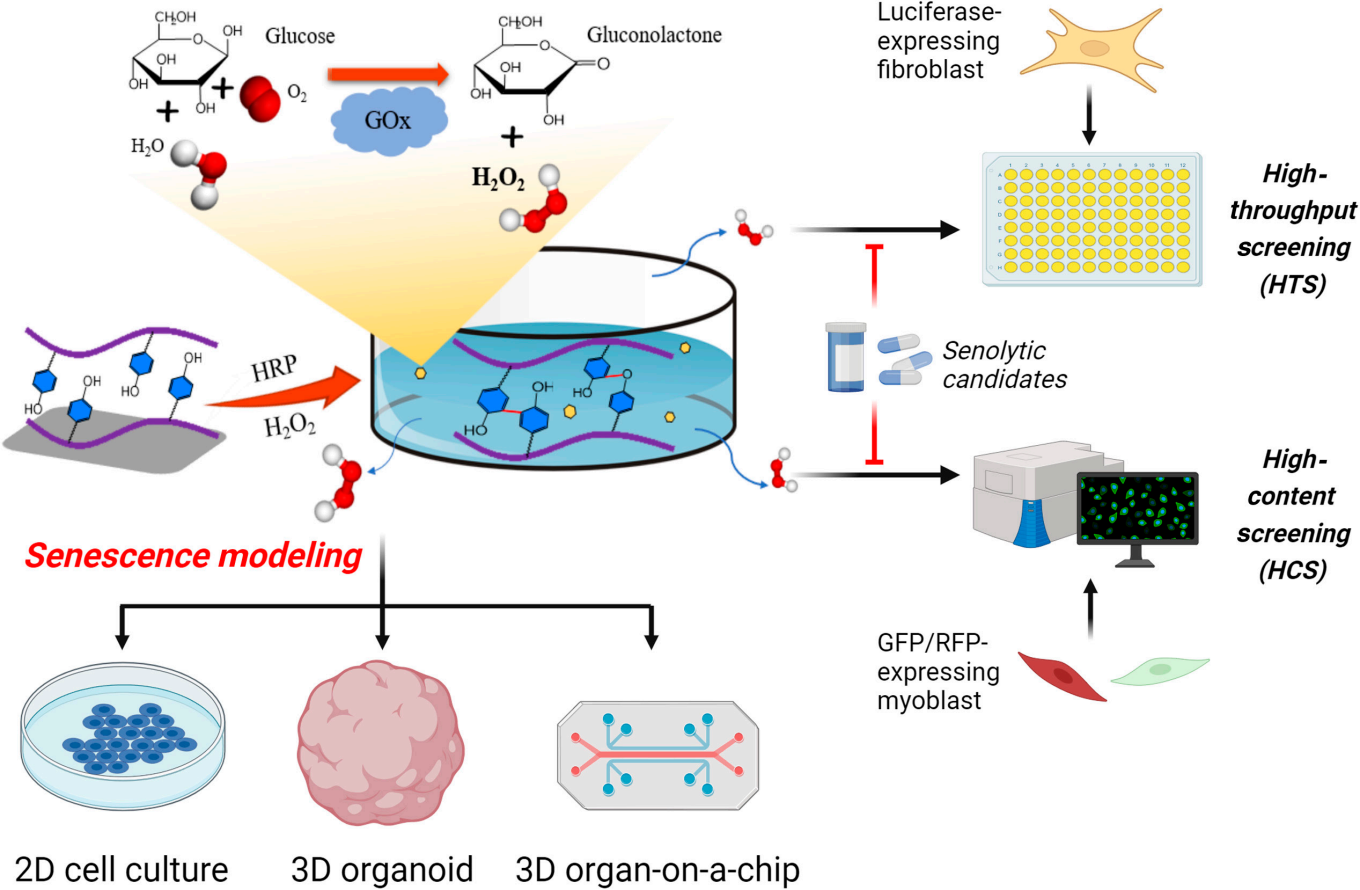
Schematic illustration of H_2_O_2_-releasing hydrogel for developing aging models in 2D cell culture and 3D organ models and establishing reporter-based senolytics screening systems for aging research. Created in BioRender. Wei (2025). https://BioRender.com/e72p321.

Herein, we developed an H_2_O_2_-releasing hydrogel (HRH) model to effectively induce cellular senescence in 2D cell culture of myoblasts and fibroblasts, as well as 3D organ models of brain-on-chips and brain organoids. Meanwhile, we have proposed 2 reporter-based senolytics screening systems via HRH, which present great potential for the treatment of aged disorders (Fig. 1). Collectively, this reliable senescence induction technology will help refine model accuracy of aged human “avatars” and better guide the development of anti-geronic therapies.

## Materials and Methods

### Materials

Gelatin (Gtn, type A from porcine skin, less than 300 bloom), horseradish peroxidase (HRP: type VI, salt-free, 250 to 330 units/mg solid), hydrogen peroxide (H_2_O_2_, 30 w/v % in H_2_O), 3-(4-hydroxyphenyl) propionic acid, 1-ethyl-3-(3-dimethylaminopropyl)-carbodiimide (EDC), *N*-hydroxysuccinimide (NHS), and deuterium oxide (D_2_O) were purchased from Sigma-Aldrich (Saint Louis, MO, USA). Dimethylformamide (DMF) was purchased from Junsei (Tokyo, Japan). The chemical reagents were used as obtained without further purification.

### Preparation of HRH

The GH polymer was synthesized with EDC/NHS as the coupling reagent mixed in the solvent with DMF and D_2_O in sequence, using the same method previously described [28]. HRH was prepared by simply mixing 2 types of solutions (Sol A and Sol B) as previously reported [29]. Briefly, to fabricate solution A, HRP (0.01 mg/ml) and glucose oxidase (GOx: 0 to 6 U/ml) were dissolved in Dulbecco’s phosphate-buffered saline (DPBS), followed by mixing with a 6.25 wt % GH solution (volume ratio of GH/HRP/GOx = 8:1:1). To prepare solution B, 6.25 wt % GH was dissolved in H_2_O_2_ (0.2 wt %) and DPBS (volume ratio GH/H_2_O_2_/DPBS = 8:1:1). To fabricate the HRH, solutions (A and B) were gently mixed in a volume ratio of A/B = 1:1 and transferred into inserts (Falcon, NY, USA) in a 24-well plate.

### Quantification of H_2_O_2_ released from HRH

To analyze the release behavior of H_2_O_2_ from the GH hydrogel, the amount of H_2_O_2_ released from the hydrogel was quantified using a Quantitative Peroxide Assay Kit (Pierce, Rockford, IL, USA) and the ferrous ion (Fe^2+^) oxidation xylenol orange assay, as previously described [30]. Briefly, 200 μl of GH hydrogel was prepared in an insert for a 24-well plate and incubated with 500 μl of Dulbecco’s modified Eagle’s medium (DMEM). At predetermined time points, 20 μl of each medium was added to a 96-well plate. A 200-μl aliquot of each assay kit was added to the collected samples and incubated at room temperature (RT) for 15 min. Absorbance was measured at 595 nm using a multimode microplate reader (BioTek Instruments, Inc., VT, USA). A calibration curve was generated using the difference in absorbance between the samples and blank solutions.

### p16-luc cells extraction

The p16^LUC^ transgenic mice [31] were maintained as previously described [32], and all animal procedures were performed according to the protocol approved by the Institutional Animal Care and Research Advisory Committee at the Sungkyunkwan University School of Medicine Laboratory Animal Research Center. Procedures aligned with the regulations of the Institutional Ethics Committee (SKKUIACUC2020-09-52-1). The p16-luc lung fibroblasts were harvested from p16^LUC^ mice according to an established protocol [33]. Briefly, lung fragments of approximately 1 cm^2^ were harvested from euthanized p16^LUC^ mice, cut into 1-mm pieces, and digested to white sticky fibers. DMEM/F12 media with 15% fetal bovine serum (FBS) and 1× antibiotic/antimycotic were added and centrifuged at 524 *g*. Pellets were resuspended in 10 ml of culture media and placed in a tissue culture incubator at 37 °C, 5% CO_2_, and 3% O_2_. Fourteen days after isolation, cells were harvested and plated onto a new plate at 5 × 10^5^ cells/plate of Eagle’s minimum essential medium (EMEM) with 15% FBS, 1× penicillin/streptomycin (PS), nonessential amino acids, and sodium pyruvate.

### Cell culture

C2C12 myoblasts were cultured in DMEM containing 10% FBS and 1% PS. WI38 fibroblasts were cultured in MEM containing 10% FBS and 1% PS. C2C12/GFP(CMV) and C2C12/RFP(CMV) stable cell lines were purchased from Cellomics Technology (Halethorpe, MD, USA) and cultured in DMEM containing 10% FBS and 1% PS. p16-luc lung fibroblast cells were cultured in EMEM containing 15% FBS, 1% PS, 1% MEM non-essential amino acid solution (100×), and 1 mM sodium pyruvate. All cells were incubated under standard conditions (37 °C and 5% CO_2_).

### Validation and optimization of senescence induction in 2D cell culture by HRH

To validate and optimize the effects of senescence induction, C2C12 myoblasts were divided into several groups, including Ctrl, GOx, and H_2_O_2_ groups. HRH with different GOx concentrations was prepared 24 h before cell seeding, supplying 0.5 ml of culture media with 4.5 g/l glucose. Cells (4 × 10^3^) were seeded in each well in all groups, supplying 1 ml of culture media. The inserts containing HRH were placed above the cells in the corresponding wells of the GOx groups. For the Ctrl and GOx groups, culture media was refreshed every 2 days; for the H_2_O_2_ group, cells were treated with H_2_O_2_ at 150 nM for 2 h and the culture media was refreshed every day after H_2_O_2_ treatment. Senescence induction was evaluated by senescence-associated β galactosidase (SA-β-Gal) activity analysis and γH2AX foci immunofluorescent staining.

### SA-β-Gal activity analysis

For SA-β-Gal activity evaluation, cells were stained for SA-β-Gal following the manufacturer's protocol (Cell Signaling Technology, Danvers, MA, USA) and stained for the β-Gal substrate according to the manufacturer's protocol (Thermo Fisher Scientific, Rockford, IL, USA). To quantify SA-β-Gal activity, the cells were counterstained with DAPI to visualize the nuclei. The percentage of senescent cells was calculated by dividing the total number of senescent cells by the total number of cells.

### CellBrite membrane staining

The cell membrane was stained following the manufacturer’s protocol (Biotium, Fremont, Bio-Rad, CA, USA), and the nuclei were stained with DAPI. To quantify the morphological changes in cells and their nuclei, the areas were compared using ImageJ software.

### Cell cycle analysis

Cells were harvested by centrifugation and fixed with ice-cold 70% ethanol (−20 °C) for 1 h at 4 °C. Prior to flow cytometry analysis, the fixed cells were repelleted by resuspension and permeabilized in DPBS containing 0.25% Triton X-100 at 4 °C for 15 min, then resuspended at a concentration of ~10^5^/ml in DPBS containing 20 μg/ml of propidium iodide and 10 μg/ml of RNase A. After the cells were incubated in the dark for 30 min, cell cycle profile analysis was carried out on 10,000 cells with a fluorescence-activated cell sorter (FACSAria, BD Biosciences, NJ, USA), and the results were analyzed using the FACSDiva software module (Becton Dickinson).

### Establishment of brain-on-chips

Brain-on-a-chip was established by coculture of neurons and astrocytes in the microfluidic platform as previously described [34]. Human neural progenitor cells (ReNcell VM, Millipore) were transduced via the lentiviral constructs with stable GFP fluorescent signal, seeded into the angular chamber at 2 × 10^4^ cells per device with 20% Matrigel in DMEM/F12 (Life Technologies, Grand Island, NY) media supplemented with 2 mg of heparin (STEMCELL Technologies, Vancouver, Canada), 2% (v/v) B27 neural supplement (Life Technologies, Grand Island, NY), and 1% (v/v) PS/amphotericin-B solution (Lonza, Hopkinton, MA). The devices were incubated at 37 °C supplied with 5% CO_2_ for 3 weeks to complete the differentiation. Afterward, HRH was loaded into the central chamber. H_2_O_2_ was directly administrated to cells in an angular chamber. For the Ctrl and GOx group, media was changed every 3 to 4 days. For the H_2_O_2_ group, media was changed with 10 μM H_2_O_2_ every day.

### Brain organoid culture

Human induced pluripotent stem cell (hiPSC) lines (CMC-hiPSC-011) were obtained from the Korea National Stem Cell Bank. hiPSCs were cultured in mTeSR1 medium (STEMCELL Technologies, Vancouver, BC, Canada) on Matrigel (Corning)-coated cell culture plates. The hiPSC colonies were manually passaged at 70% confluence and maintained at 37 °C in humidified air with 5% CO_2_. hiPSC-derived cerebral organoids were achieved using the STEMdiff Cerebral Organoid Kit (STEMCELL Technologies, catalogue number 08570). hiPSCs were differentiated into brain organoids using the STEMdiff Cerebral Organoid Kit product manual. Brain organoid formation was based on a formulation published previously [35,36]. A total of 9,000 cells were plated in each well of an ultralow-binding 96-well plate (Corning) in EB Formation Medium containing 10 μM Y-27632 (STEMCELL Technologies). EBs were fed every other day for 5 days and then transferred to low-adhesion 24-well plates with induction medium for 3 days. After neuroepithelium formation, the tissues were transferred to droplets of cold Matrigel and transferred to low-adhesion 6-well plates with expansion medium. After 3 days of stationary growth, the medium was replaced with Maturation Medium. The organoids were placed on an orbital shaker in a 37 °C incubator. This final maturation medium was replaced once every 3 days to day 20.

### Senolytics drugs screening

Senescence of p16-luc and C2C12-GFP cells was induced by the HRH model under optimal conditions. High-throughput screening (HTS) data were obtained from Synergy Neo (BioTek). Senolytic activity was calculated using the following equation: Senolytic activity = 1/log_10_ (luciferase intensity in p16-luc cells). For high-content screening (HCS), data were obtained using NIS-Elements (Nikon). Senolytic activity was calculated using the following equation: senolytic activity = 1/log_10_ (C2C12-GFP cell numbers).

### Statistical analysis

All experiments were repeated at least 3 times, with identical or similar results. Data represent biological replicates. Adequate statistical analyses were performed for each assay. The data met the hypotheses of the statistical tests described in each experiment. Statistical significance of differences was calculated using the 2-tailed paired or unpaired Student’s *t* test between 2 groups, with one-way analysis of variance (ANOVA) among 3 or more groups using GraphPad Prism 8.4.3 (GraphPad Software Inc., San Diego, CA, USA). *P < 0.05, **P < 0.01, ***P < 0.001, and ****P < 0.0001 were considered statistically significant.

## Results

### Characteristics of the HRH model and controlled H_2_O_2_ release

**Fig. 2.**
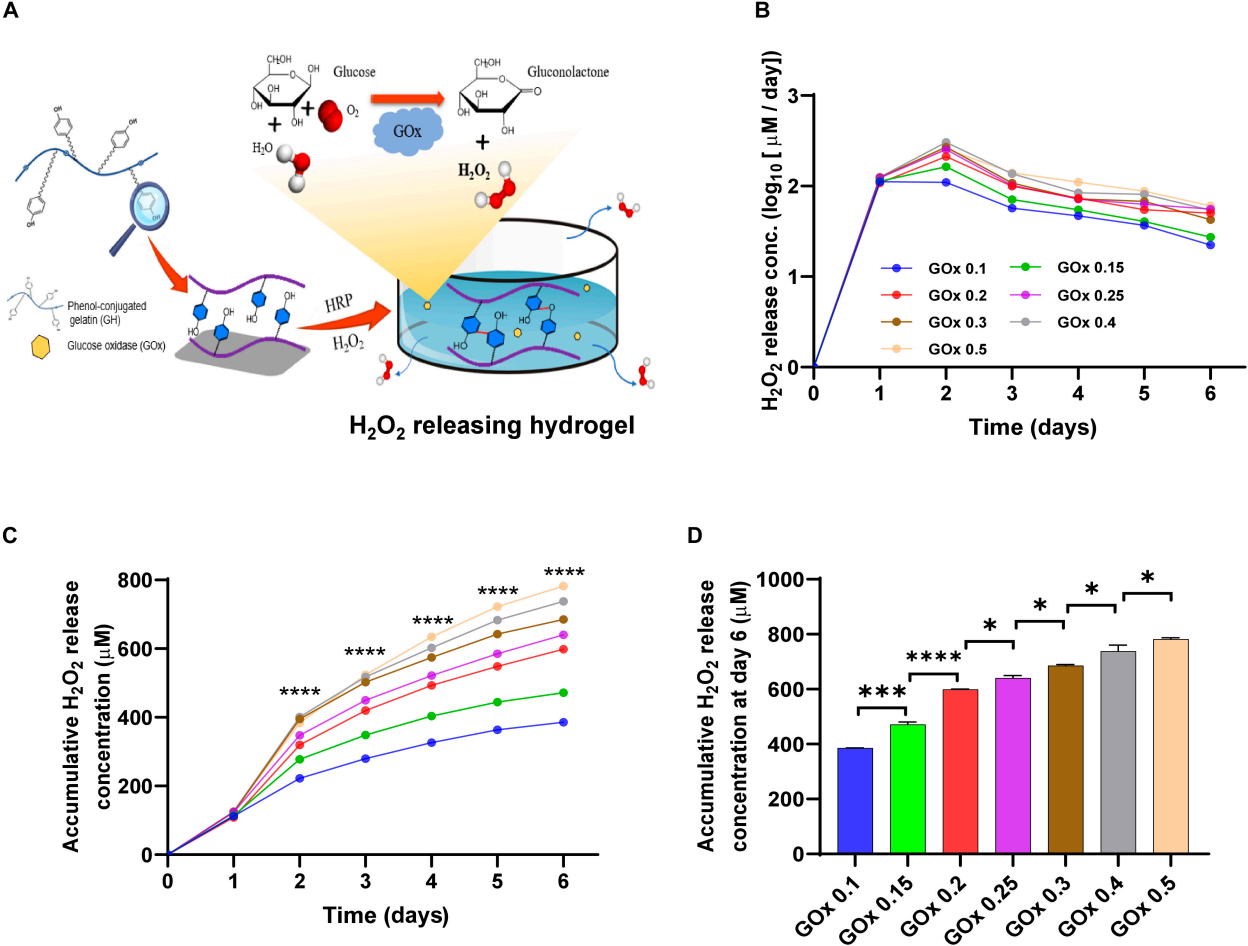
HRH synthesis and controlled H_2_O_2_ release behavior. (A) Schematic representation of HRH synthesis through dual enzyme-mediated crosslinking reaction. Glucose in the media infiltrates into the hydrogel matrix where it reacts with GOx to release H_2_O_2_. (B to D) In vitro H_2_O_2_ release behavior from HRH at various GOx concentrations with quantifications. All quantitative data are shown as mean ± SEM (*n* = 3).

In the previous study, we developed a Gtn-g-hydroxyphenyl propionic acid (GH) hydrogel that stimulated vascular cell activity and neovascularization without compromising cytotoxicity [30]. The GH hydrogel was fabricated using a dual enzyme-mediated crosslinking procedure with HRP and GOx as H_2_O_2_-producing sources, demonstrating the peculiarity that H_2_O_2_ release could be altered by regulating GOx concentrations. The H_2_O_2_ release behavior is designed to be accurately modulated with the same crosslinking reaction and optimized to stably induce cellular senescence in vitro. Of note, in our prior study, glucose was used as an essential substrate for generating H_2_O_2_ as well as a component in the synthesis of HRH. However, for senescence induction, substantial glucose is required to produce adequate H_2_O_2_ and thereby induce sufficient oxidative stress. Therefore, we optimized the procedure by removing glucose from GH synthesis as per the original design, instead feeding culture media with high glucose concentrations (4.5 g/l) directly to the HRH to ensure long-term and sustained H_2_O_2_ generation (Fig. 2A). We subsequently investigated the modulation of GOx concentrations (0 to 4 U/ml) on H_2_O_2_ release using ferrous (Fe) ion oxidation xylene orange assay. Enhancing GOx concentrations facilitated an incremental increase in H_2_O_2_ release over 6 days (Fig. 2B to D), demonstrating that our HRH provides a dynamic hydrogel matrix with precisely controllable and consistent properties of H_2_O_2_ release.

### Optimization of HRH-induced cellular senescence

**Fig. 3.**
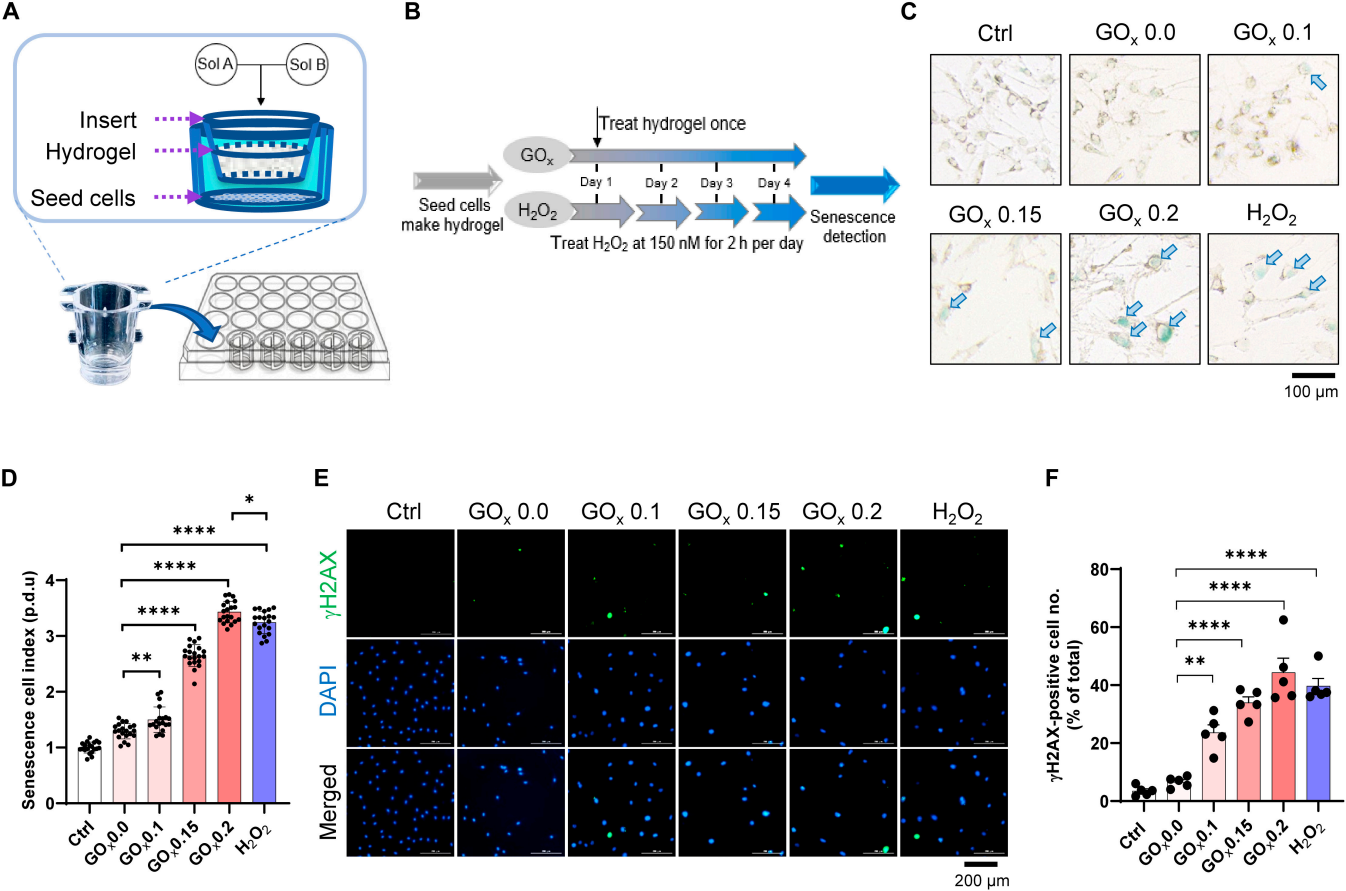
Development and optimization of cellular senescence by HRH model. (A) Conceptual figure represents developed application of HRH model in a 24-well plate. (B) Schematic representation of senescence induction protocols. (C) Senescence induction effect is evaluated by SA-β-Gal staining with quantitative results (D). Scale bar, 100 μm. (E) Further confirmation is conducted by γH2AX staining with quantitative results (F). Scale bar, 200 μm. The quantitative results are shown as mean ± SEM. Statistical significance is denoted as *P < 0.05, **P < 0.01, ****P < 0.0001.

To evaluate the induction effect of HRH in C2C12 myoblasts, HRH was positioned within the inserts, which were set above cells that adhered at the bottom of 24-well plates (Fig. 3A), thereby allowing the released H_2_O_2_ to penetrate the culture medium through the permeable bottom of inserts to cells. This strategy allows HRH to react with glucose without interfering with cell proliferation, while preventing uneven senescence induction or cell death induced by directly attaching cells within or on the hydrogel. While determining optimized conditions for induction, we observed that growth-arrested cells displayed enlarged morphologies and granule accumulation in the GOx and H_2_O_2_ groups after 4 days (Fig. 3B), which resemble senescent characteristics. To confirm our hypothesis, C2C12 myoblasts were subjected to SA-β-Gal staining and validated using γH2AX staining (Fig. 3C and E). Strikingly, cells in the GOx groups and H_2_O_2_ group manifested positive signatures, indicating the efficacy of our HRH models in triggering senescence. In addition, the elevated percentage of senescent cells in GOx groups was observed in a dose-dependent manner, with the optimal concentration (GOx 0.2) even giving better results compared to that of the H_2_O_2_ group (Fig. 3D and F).

### Validation of HRH-induced cellular senescence

**Fig. 4.**
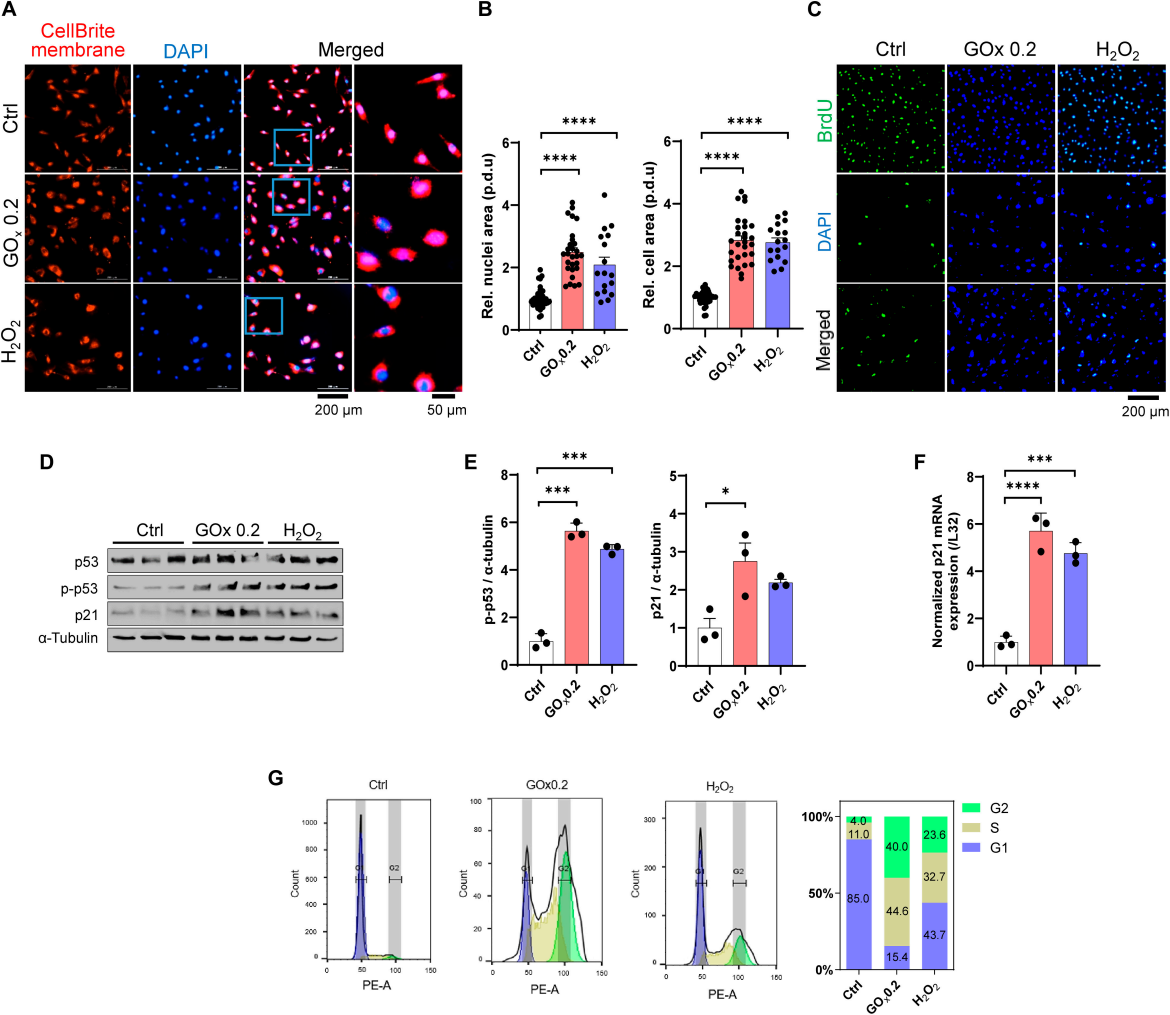
Validation of HRH-induced cellular senescence. (A) Fluorescent images of cell membrane and nuclei staining representing morphology alterations of senescent cells. Scale bar, 200 μm. Magnified scale bar, 50 μm. (B) Quantification of the area of cells and nucleus. (C) BrdU incorporation assay indicating cell proliferation. Scale bar, 200 μm. (D) Western blot showing increased expression level of senescence markers with quantitative results (E). (F) qRT-PCR showing up-regulated transcription level of senescence markers. (G) Flow cytometry analysis evaluating cell cycle arrest by propidium iodide staining and stacked bar graphs summarizing the percentage of cells at each stage of cell cycle. The quantitative results are shown as mean ± SEM. Statistical significance is denoted as *P < 0.05, ***P < 0.001, ****P < 0.0001.

For further validation, immunostaining was conducted to visualize the senescent features, including morphological alterations and cell proliferation halt [37]. GOx 0.2-treated cells exhibited an evident enlarged size of cellular structure and nucleus (Fig. 4A and B and Fig. S1), along with a marked stagnation of cell proliferation evidenced by 5-bromo-2′-deoxyuridine (BrdU) incorporation (Fig. 4C). In addition, senescent cells are arrested at the G1/S phase, yet remaining metabolically active [38], which is positively linked to the activation of p53, p21, and p16. In this regard, we evaluated senescence-associated biomarker alterations in different groups and observed higher expression levels of p21 and phospo-p53 in GOx 0.2-treated cells compared to those in the other 2 groups (Fig. 4D and E). Likewise, overexpression of p21 was observed in the GOx 0.2 group, indicating a high degree of senescent marker transcription (Fig. 4F). To consolidate this observation, cell cycle arrest was directly evaluated using flow cytometry (Fig. 4G). A higher proportion of cells in the GOx 0.2 group arrested in the S phase in comparison to H_2_O_2_, which provided compelling evidence of the induction for senescence. We further verified the feasibility of using different cell lines, p16 fibroblasts (Fig. S2). A similar induction efficiency reaffirmed the effectiveness and wide adaptability of our HRH model.

### HRH induces cellular senescence in brain-on-chips and brain organoids

**Fig. 5.**
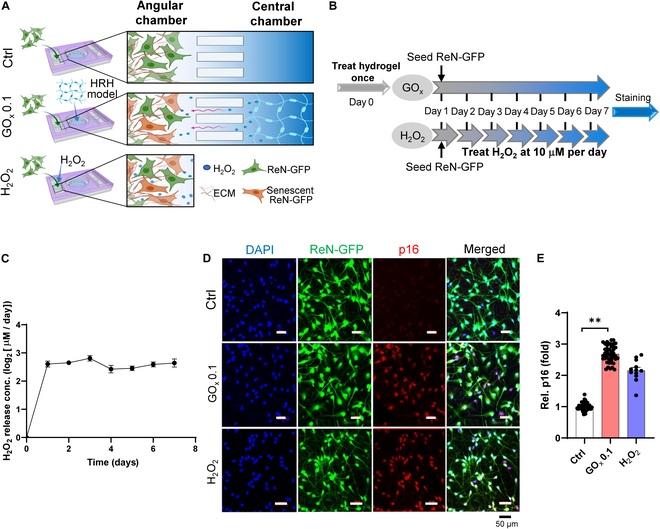
HRH-induced cellular senescence in brain-on-a-chip. (A) Conceptual figures for 3D layouts and treatment in brain-on-a-chip for different groups. (B) Schematic representation of senescence induction protocols. (C) H_2_O_2_ release behavior from HRH in brain-on-a-chip. (D) Immunofluorescent staining for ReN-GFP cells, senescent marker p16, and DAPI, with quantitative results (E). Scale bar, 50 μm. The quantitative results are shown as mean ± SEM. Statistical significance is denoted as **P < 0.01. ECM, extracellular matrix.

To construct aged human “avatars”, we next employed our HRH system to promote cellular senescence and develop the aged human brain models in microfluidics. To this end, we first cultured neuro-progenitor cells in the angular side of microfluidic device, which further differentiated into neurons and astrocytes in 3 weeks (Fig. 5A). Upon the completion of differentiation, we added cell culture media supplemented with HRH with GOx 0.1 or H_2_O_2_ (10 μM) into the central chamber to allow the continuous release of H_2_O_2_ from the central to the angular side through the microchannels connecting both chambers. For the ctrl counterpart, we only added the culture media to the central compartment. Unlike the H_2_O_2_ group, which required daily treatment, the GOx group underwent only a single treatment, demonstrating a simpler procedure (Fig. 5B). To validate the reliability of our HRH for the sustained release of H_2_O_2,_ we measured H_2_O_2_ concentration in the central chamber and found that HRH with GOx 0.1 released H_2_O_2_ at 6.13 ± 0.5 μM per day for a week (Fig. 5C). Results from immunofluorescent staining displayed significant increase on p16 expression compared to that of the Ctrl group (Fig. 5D), and even a slightly better effect than that of the H_2_O_2_ group at 10 μM per day (Fig. 5E).

**Fig. 6.**
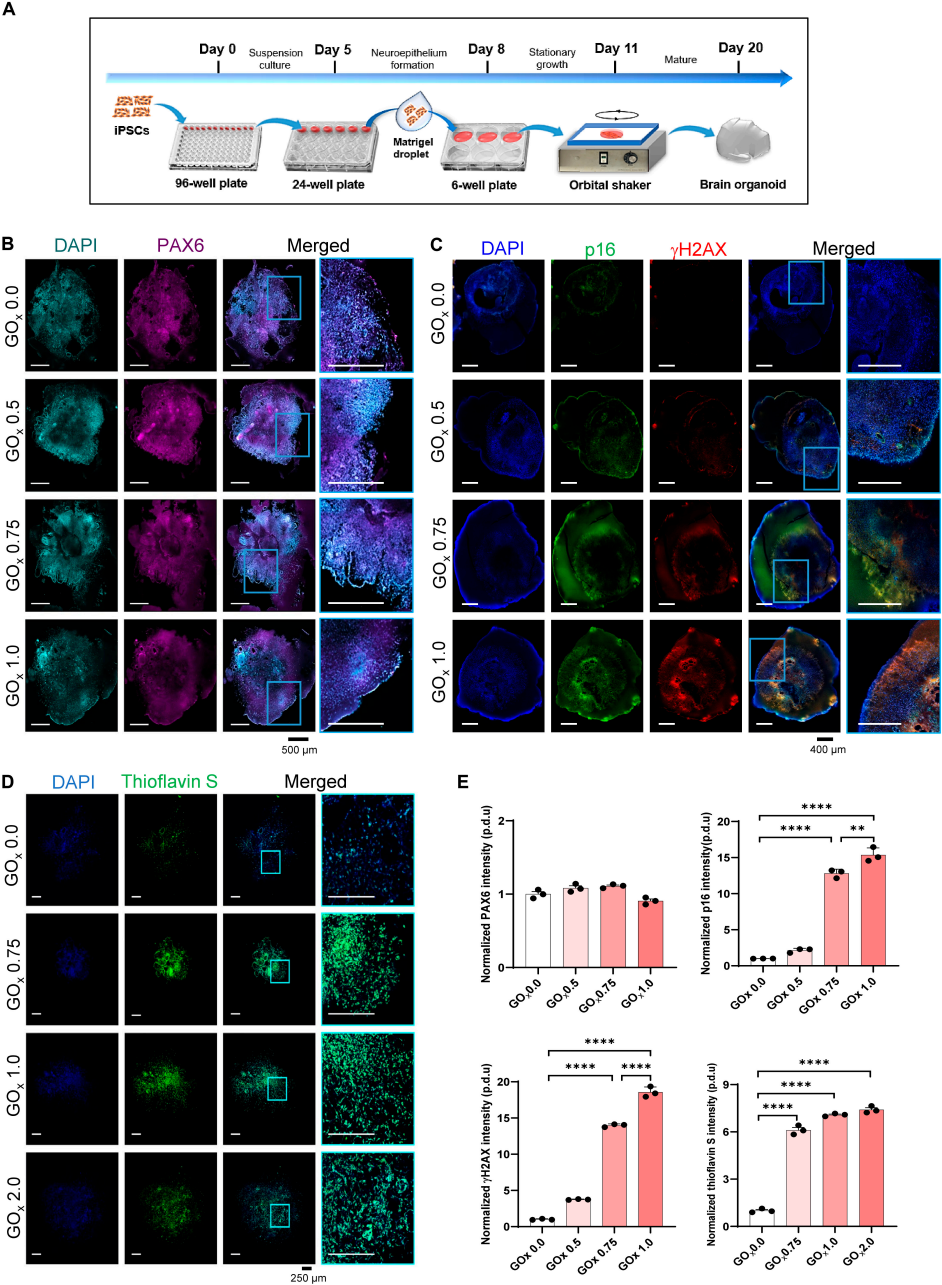
HRH-induced cellular senescence in brain organoid. (A) Timeline of the protocol for generating brain organoids. (B) Representative images of brain organoids with neural progenitor cells marker PAX6 and DAPI at 20 days of differentiation. Scale bar, 500 μm. (C) Immunofluorescent staining of brain organoids for senescence markers p16 and γH2AX counterstained with DAPI. Scale bar, 400 μm. (D) Immunofluorescent staining for fibrillar amyloid aggregation marker thioflavin S and DAPI. Scale bar, 250 μm. (E) Quantification of foci intensity of PAX6, p16, γH2AX, and thioflavin S normalized with DAPI. The quantitative results are shown as mean ± SEM. Statistical significance is denoted as **P < 0.01, ****P < 0.0001.

Aside from brain-on-chips, we next assessed the efficacy of brain organoids. For validation, brain organoids were established by this process (Fig. 6A). As expected, the neural progenitor cell marker PAX6 was highly expressed during organoid differentiation, indicating successful brain organoid establishment (Fig. 6B). Dual fluorescent staining revealed that the senescence markers, p16 and γH2AX, increased as the GOx concentration rose, confirming that our HRH was able to induce senescence in brain organoids (Fig. 6C). Meanwhile, results from thioflavin S staining demonstrated that increased GOx concentration could also aggregate in fibrillar amyloid deposition, which was a crucial incentive of multiple neurological disorders such as Parkinson’s disease and Alzheimer's disease (Fig. 6D). Notably, senescence and amyloid deposition levels were critically altered in organoids when GOx concentrations exceeded 0.75 U/ml (Fig. 6E).

### Senolytics screening via HTS and HCS

**Fig. 7.**
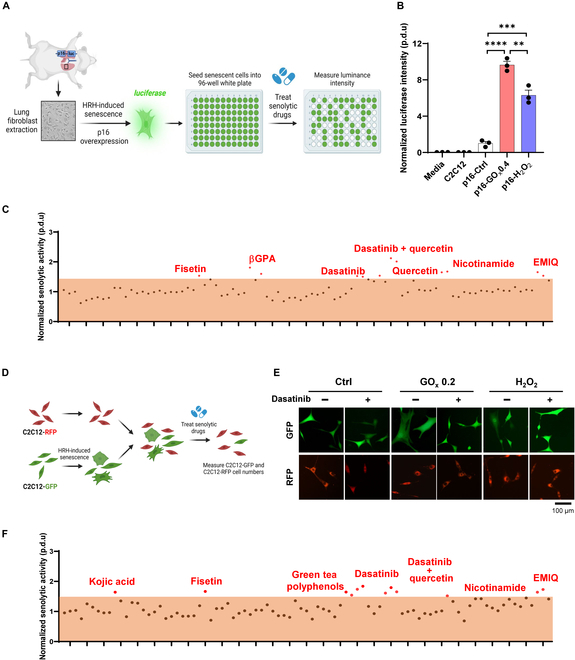
Senolytics screening via HTS and HCS. (A) Schematic diagram illustrating the experimental system used to screen senolytics in p16-luc lung fibroblasts using HTS. Created in BioRender. Wei (2025). https://BioRender.com/e72p321. (B) Luciferase intensity of senescent p16-luc fibroblasts induced by HRH and H_2_O_2_ and normalized with normal cells. The results are shown as mean ± SEM. Statistical significance is denoted as ****P < 0.0001. (C) Scatter plot representing the normalized senolytics activity obtained from 29 potential senolytics by measuring luminance intensity. Red spots representing highest activities are marked with corresponding names. (D) Schematic diagram illustrating the experimental system used to screen senolytics in C2C12-GFP and C2C12-RFP cells using HCS. Created in BioRender. Wei (2025). https://BioRender.com/e72p321. (E) Fluorescent images on normal and senescent cells treated with or without anti-senescent drugs (i.e., dasatinib) indicating no cytotoxicity on normal cells and potential senolytic effects on senescent cells. Scale bar, 100 μm. (F) Scatter plot representing the normalized senolytics activity obtained with 29 potential senolytics by measuring C2C12-GFP cell number. Red spots representing highest activities are marked with corresponding names.

To discover potential senolytics, conditions were set up using HTS on p16-luc cells, a mouse lung fibroblast cell line harvested from p16^LUC^ mice (Fig. 7A). After treatment with HRH, the expression level of p16 increased, thereby enhancing luciferase intensity. Senolytic activity was determined by detecting the luminance following the administration of certain substances. As a proof of concept, we demonstrated that the luciferase intensity of senescent p16 fibroblasts, especially that induced by HRH, was markedly and statistically increased compared to the p16-Ctrl group (Fig. 7B). We then compared senescence inducers to drugs with potential senolytic properties as well as promising drugs that may regulate senescence progression (total of 29 candidates), using the HRH model-induced senescent p16-luc cells (Fig. 7C). Potential drugs with the highest senolytic activity are highlighted in red and marked with their individual names. The side cytotoxic effects of these drugs were identified in normal cells treated with each drug independently (Fig. S3).

In an independent manner, another senolytic screening platform was established using HCS in C2C12-GFP and C2C12-RFP cells. C2C12-GFP cells were first induced to senescence the HRH model and then mixed with normal C2C12-RFP cells in a ratio of 3:1 in 96-well plates (Fig. 7D). Twenty-four hours after administration, the numbers of C2C12-GFP and C2C12-RFP cells were counted. Fluorescent images of normal and senescent cells treated with or without senolytics were assessed to verify the senolytic effect of the drugs by eliminating the enlarged senescent cells (Fig. 7E). Here, dasatinib was used as an example and compared with those in untreated cells; dasatinib-treated cells showed elimination of senescent C2C12-GFP cells, but no difference in C2C12-RFP cells, in either RFP intensity or cell count, which indicates an effective senolytic effect of the drug. Then, variations in the cell number of C2C12-GFP were measured to assess senolytic activities among the 29 candidate drugs. Strikingly, several of them exhibited robust efficiency in removing senescent cells (Fig. 7F). Compared to that of HTS, several others also demonstrated certain senolytic effects, including kojic acid and green tea polyphenols.

## Discussion

Evidence from the current study emphasizes that reducing senescent cell burden could prolong health and lifespan to a large extent, even late in life. This highlights the necessity of developing a reliable model of replicating cellular senescence in the context of aging research. However, traditional methodologies, typified by employing H_2_O_2_ to induce senescence, present notable limitations, mainly manifested in the heterogeneity and instability caused by transitory H_2_O_2_ exposure, as well as apoptosis resulting from acute oxidative stress. To overcome these constraints and to follow more in line with the etiology of age-related disorders, we constructed an HRH that continually releases H_2_O_2_, utilizing dual enzymatic crosslinking reactions to induce chronic cellular senescence via prolonged oxidative stress. Building on our previous design [30], we replaced glucose in the hydrogel components with a high-glucose medium, enabling sustained H_2_O_2_ release and overcoming the spatial and operational constraints associated with traditional induction methods. While the material itself may not represent a groundbreaking advancement, this study substantially expands the usability and applicability of our hydrogel system, particularly in the context of aging research. This refinement bridges critical conceptual and practical gaps, offering a robust and physiologically relevant platform for exploring cellular senescence. Furthermore, we demonstrated that H_2_O_2_ production from the HRH can be precisely modulated by adjusting GOx concentration, thereby allowing for the fine-tuning of oxidative stress levels to suit the tolerance of specific conditions. This tunable feature not only broadens the system’s versatility but also facilitates customized senescence modeling and supports broader applications in high-throughput and long-term studies. In this experiment, we tested H_2_O_2_ production under various GOx settings over the course of 0 to 6 days, demonstrating controlled and persistent release. Senescent induction was next evaluated with C2C12 myoblasts. After optimization, full-scale evaluations were employed, including analyses on senescent characteristics, senescence-associated biomarker variations, and intuitive mitotic arrest. Overall, these results reinforced the conclusion that the HRH induced senescence in a stable and consistent manner, being evidently superior to the traditional senescence induction strategy of treating H_2_O_2_ directly in terms of both technique and efficacy. Simultaneously, our HRH also showed effective inducibility in fibroblasts, indicating its potential and broad applicability in senescence induction across different cell lines. Altogether, we believe that the HRH can be employed as a stable, effective, and widely adopted technology for inducing senescence in vitro.

While it is noteworthy to mention that beyond the use of H₂O₂ as the primary inducer of senescence, incorporating alternative agents such as DNA-damaging compounds (e.g., etoposide and bleomycin) [39], chemical oxidants (e.g., tert-butyl hydroperoxide) [40], and pro-inflammatory cytokines (e.g., interleukin-6 and tumor necrosis factor-α) [41] could markedly expand the utility of our hydrogel system. These substances could simulate distinct senescence pathways, such as genomic instability, acute oxidative stress, or senescence-associated secretory phenotype (SASP)-related inflammation, respectively. For instance, DNA-damaging agents could model the persistent genomic instability observed in aging-related disorders, while cytokine-releasing hydrogels could recapitulate the pro-inflammatory microenvironment characteristic of the SASP. By integrating these alternative pathways, the hydrogel platform could evolve into a multifaceted system capable of mimicking the complex and interconnected processes underlying cellular aging and its associated diseases, facilitating further aging investigations. Nevertheless, the controlled and tunable delivery of H_2_O_2_ remains the cornerstone of this study, providing a physiologically relevant and scalable method for senescence induction.

Currently, 2D cell culture is a commonly used in vitro tool to improve our understanding of cell biology, disease pathophysiology, the mechanisms of medication, protein production, and tissue engineering development. The advantages include a simple and low-cost upkeep, as well as the ability to perform functional tests. Unfortunately, the historical emphasis on cells in biological research has made it difficult to address challenges specific to our comprehension of human physiology and pathology [42]. Under these circumstances, precision medicine has emerged as an innovative strategy for personalizing disease prevention and therapy by considering variations in people’s genes, environments, and lifestyles. To realistically simulate disease progression in vitro, 3D human “avatars”, namely, OOC and organoids, have been recently developed. These 3D microphysiological systems have a similar composition and architecture to primary tissue, manipulating niche components and gene sequences to model multiple functional organs in vitro [43], thereby allowing researchers to study advanced human diseases, from early development through to aging. However, subject to the elimination of DNA methylation, a critical regulator of biological age across the entire human lifespan and even during development, iPSC-derived 3D organs might not comprehensively replicate epigenetic signatures during the aging process. Hence, generating stable cellular senescence on the 3D organ models is obligatory for aged human “avatar” establishment and prospective aging research. Based on the drawbacks of traditional approaches, our HRH system is thought to have great potential for reproducing epigenic modulation and aging progression on the OOC and organoids due to the robust fluidity of hydrogel before solidification, controllable H_2_O_2_ release behavior, and effective senescence induction. In this experiment, we attempted to adapt HRH to 3D brain-on-chips and brain organoids. Synergy results showed that HRH was utilized to efficiently generate senescence by variation in the expression of senescent biomarkers β-Gal and p16, and the DNA damage marker γH2AX. Moreover, with an increase in GOx concentration, the expression of neuropathy-associated factors was enhanced, providing compelling evidence of its substantial potential in establishing aged human models in vitro and mimicking aging progression in vivo.

Another crucial application of HRH is the discovery of senolytics for anti-aging therapies. Senolytics are a family of tiny compounds that are being studied to determine whether they can selectively eliminate senescent cells and enhance human health [1]. The pharmacological clearance of senescent cells with senolytics improves various age-related disorders in model organisms. Senolytics have a wide range of potential applications, with preclinical research indicating that they could help treat more than 40 ailments, including age-related dysfunction [44]. To date, approaches to identifying senolytics include bioinformatic analysis to identify senescent cell anti-apoptotic pathways [20], structure–activity relationship studies to determine the critical features of senolytics involved in potency and specificity [45], and SA-β-gal expression to evaluate their ability to remove senescent cells [46]. However, there are few effective platforms for simple and extensive senolytic screening. Therefore, we set out to identify more senolytic drugs that would allow the discovery of vulnerabilities in senescence and provide novel therapeutic options for age-related disorders. In this study, we designed 2 robust screening systems based on HRH for discovering senolytic activities and assessed them with 29 compounds using HTS and HCS. Based on the comparison between the 2 strategies, 5 compounds, including fisetin, dasatinib, quercetin, nicotinamide, and enzymatically modified isoquercitrin, were shown to have persistent and highly effective senolytic properties but no cytotoxicity. We also screened out other promising candidates with superior capability to eliminate senescent cells. Of note, individual evaluations of these drugs are still required in follow-up tests to clarify their optimal efficacies.

In conclusion, we have developed an HRH as a powerful tool that can effectively induce cellular senescence in 2D cell culture as well as 3D organ models, with great potential applications in anti-aging therapy. Future studies are expected to deepen our understanding of aging and develop comprehensive strategies to treat age-related diseases, thus improving human health and prolonging human life.

## Data Availability

The data that support the findings of this study are available from the corresponding author upon reasonable request.
