# Supplementary material for: Hydrogen Peroxide-Releasing Hydrogel-Mediated Cellular Senescence Model for Aging Research
Source: Biomater Res. 2025 Mar 14;29:0161. doi: 10.34133/bmr.0161 (PMC11907071; doi:10.34133/bmr.0161)
Supplement: Supplementary 1 — Figs. S1 to S3 [file bmr.0161.f1.docx]

**Supporting Information for**

**Hydrogen Peroxide-Releasing Hydrogel Mediated Cellular Senescence Model for Aging Research**

Shibo Wei^#^, Phuong Le Thi^#^, Yan Zhang^#^, Moon-young Park, Khanh Do, Thi Thai Thanh Hoang, Nyssa Morgan, Tam Dao, Jimin Heo, Yunju Jo, You Jung Kang, Hansang Cho, Chang-Myung Oh, Young C Jang*, Ki-Dong Park*, Dongryeol Ryu*

Correspondence to: [young.jang@emory.edu](mailto:young.jang@emory.edu); [kdp@ajou.ac.kr](mailto:kdp@ajou.ac.kr); [dryu@gist.ac.kr](mailto:dryu@gist.ac.kr)

**This PDF file includes:**

Methods

Figures S1 to S3

**Supplementary Experimental Section**

*Antibody*: Primary antibody: anti-p-Histone H2A.X (20E3) (Cell Signaling, 9718S, 1:200); anti-BrdU (Oncogene, NA-61-100UG, 1:100); anti-p21 (Santa Cruz Biotechnologies, SC6246, 1:300), anti-p53 (Santa Cruz Biotechnologies, SC126, 1:1000); anti-phospho-p53 (Cell Signal, 9284, 1:1000); anti-α-tubulin (GeneTex, 628802, 1:1000). anti-p16 (BD Biosciences, 554079, 1:100). The secondary antibodies were Alexa Fluor 488 goat anti-rabbit IgG (Invitrogen, A11008, 1:400), Alexa Fluor 546 goat anti-rabbit IgG (Invitrogen, A11010, 1:400), Alexa Fluor 488 goat anti-mouse IgG (Invitrogen, A11001, 1:400), Alexa Fluor 546 goat anti-mouse IgG (Invitrogen, A11003, 1:400), goat anti-mouse secondary antibody HRP (Invitrogen, G21040, 1:5000), and goat anti-rabbit secondary antibody HRP (Invitrogen, G21234, 1:5000).

*Immunofluorescence staining*: Cells in 2D and 3D cultures were fixed with 4% paraformaldehyde for 15 min. and permeabilized with 0.1% TritonX-100 for 15 min. at RT. Cells were incubated for 1 h in a blocking solution containing 3% BSA and then stained with the indicated primary antibodies for 2 h at RT in a humidified chamber. The cells were washed and incubated with secondary antibodies for 1 h at RT in a dark humidified chamber. DAPI staining was used to stain the nuclei. Images were acquired using Cytation 5 software (BioTek). Data obtained from Cytation 5 were analyzed using a Gen5^TM^ Microplate Reader and Imager Software.

*Bromodeoxyuridine (BrdU) staining*: Cells were cultured in fresh media containing BrdU at 10 μM for 4 h under standard culture conditions (37°C and 5% CO_2_). Cells were fixed with ice-cold methanol (-20°C) for 4 min at 4°C and treated with hydrochloride at 2M for 20 min at RT for DNA denaturation. Cells were treated with primary anti-BrdU antibody and Alexa Fluor 546 secondary antibody after blocking, as previously described. Images were acquired using Cytation 5 software (BioTek).

*Western blot*: Cells were lysed in RIPA buffer (pH 7.5 Tris-HCl, 150 mM sodium chloride, 0.5% sodium deoxycholate, 1% Triton X-100, 0.1% SDS, 2 mM EDTA) supplemented with phosphatase and protease inhibitor cocktail (1:200, Millipore, USA). Phosphatase inhibitors used were 5 mM sodium fluoride and 2 mM sodium orthovanadate. Western blot was conducted following standard protocols. Blots were finally visualized and analyzed using FUSION Solo X (VILBER, Collégien, France).

*RNA extraction, cDNA synthesis and Reverse Transcription PCR*: cDNA synthesis was performed following the manufacturer's protocol using the RevertAid First Strand cDNA Synthesis Kit (Thermo Scientific, IL, USA). Briefly, total RNA was isolated using TRIzol (Gibco BRL, NY, USA) and cDNA synthesis was performed using ResertAid Reverse Transcriptase (200 U/μL, Thermo Scientific, Rockford, IL, USA). The resulting cDNA was amplified using AccuPower™ PCR Premix (Bioneer, Daejeon, Korea) with the following sets of primers: L32: forward 5’ -ATGGCTCCTTCGTTGCTGC- 3’, reverse 5’ -CTGGACGGCTAATGCTGGT- 3’; p21: forward 5’ -GTACTTCCTCTGCCCT GCTG- 3’, reverse 5’ -AGAAGACCAATCTGCGCTTG- 3’. Amplification was performed for 40 cycles at 95℃ for 30 s, 55℃ for 30s, and 72℃ for 60s, followed by 55℃ for 10 min. and 72 ℃ for 7 min. The PCR products were further analyzed using a PCR Thermal Cycler Dice (TaKaRa, Seoul, Korea).

*Senolytics Candidates*: The 29 senolytic candidates lists in order: Ginsenoside Rg1(Sigma-Aldrich, [22427-39-0](https://www.sigmaaldrich.com/KR/en/search/22427-39-0?focus=products&page=1&perpage=30&sort=relevance&term=22427-39-0&type=cas_number), 20 μM); Ginsenoside Rg3 (Sigma-Aldrich, 14197-60-5, 25 μM); Kojic Acid (Sigma-Aldrich, 501-30-4, 50 μM); Oligomycin (Sigma-Aldrich, 1404-19-9, 100 nM); Antimycin (Sigma-Aldrich, 1397-94-0, 100 nM); Oligomycin & Antimycin (100 nM & 100nM); Epoxomicin (Sigma-Aldrich, 134381-21-8, 40 nM); Fisetin (Sigma-Aldrich, 528-48-3, 10 μM); 2- [2- [4-(trifluoromethoxy)phenyl]hydrazinylidene]-propanedinitrile (FCCP) (Sigma-Aldrich, 370-86-5, 100 nM); Metformin (Sigma-Aldrich, 1115-70-4, 10 μM); [β-Guanidinopropionic acid (β-GPA) (Sigma-Aldrich, 353-09-3, 5mM)](https://en.wikipedia.org/wiki/Guanidinopropionic_acid); Dimethyl 2-oxoglutarate (Sigma-Aldrich, 13192-04-6, 1mM); Ginsenoside Ra (PubChem, 83459-41-0, 25 μM); Rapamycin (Sigma-Aldrich, 53123-88-9, 500 nM); Green tea extract (Sigma-Aldrich, Y0001935, 50 μM); Green tea polyphenols (Sigma-Aldrich, [324880](https://www.sigmaaldrich.com/KR/en/product/mm/324880), 50 μM); Dasatinib (Sigma-Aldrich, 302962-49-8, 100 μM); Quercetin (Sigma-Aldrich, 849061-97-8, 25 μM); Dasatinib & Quercetin (100 μM & 25 μM); Urolithin A (Hygieia Biotech, 1143-70-0, 10 μM); Ginseng berry pomace (provided by [Amorepacific](https://www.apgroup.com/int/en/about-us/research-innovation/rnd/beauty-research-innovation/beauty-research-innovation-10.html), 100 μg/ml); Nicotinamide (Hygieia Biotech, 1094-61-7, 2mM); Bafilomycin A (Sigma-Aldrich,88899-55-2, 5 nM); Ginsenoside Re (Sigma-Aldrich, 52286-59-6, 25 μM); (-)Syringaresinol (Sigma-Aldrich, 136997-64-3, 25 μM); (+)Syringaresinol (PubChem, 1177-14-6, 25 μM); (+)-Syringaresinol (PubChem, 21453-69-0, 25 μM); Enzymatically modified isoquercitin (EMIQ, provided by [Amorepacific](https://www.apgroup.com/int/en/about-us/research-innovation/rnd/beauty-research-innovation/beauty-research-innovation-10.html), 25 μM).

**Supplementary Figures**


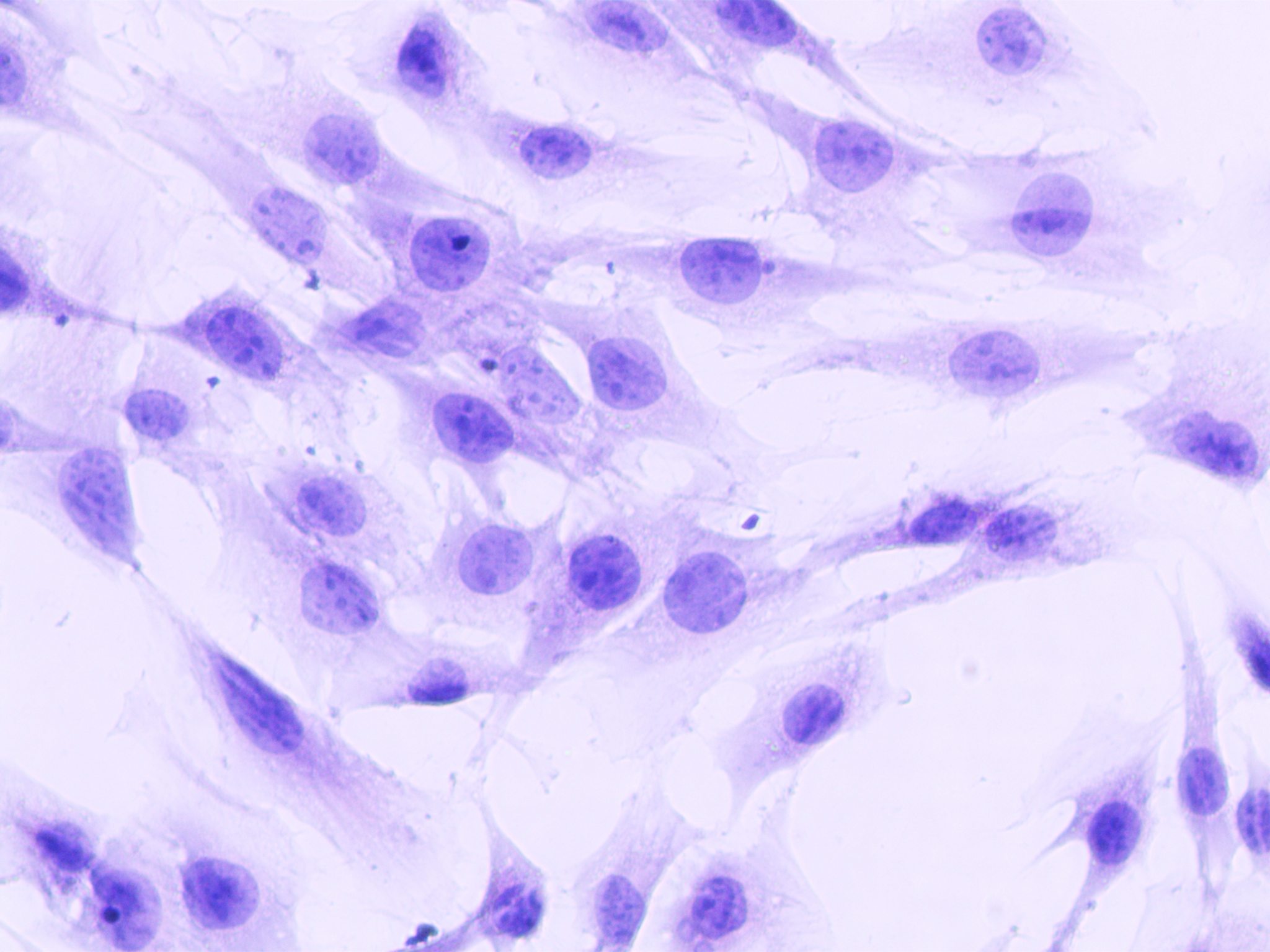


GO_x_ 0.2

Ctrl

H_2_O_2_


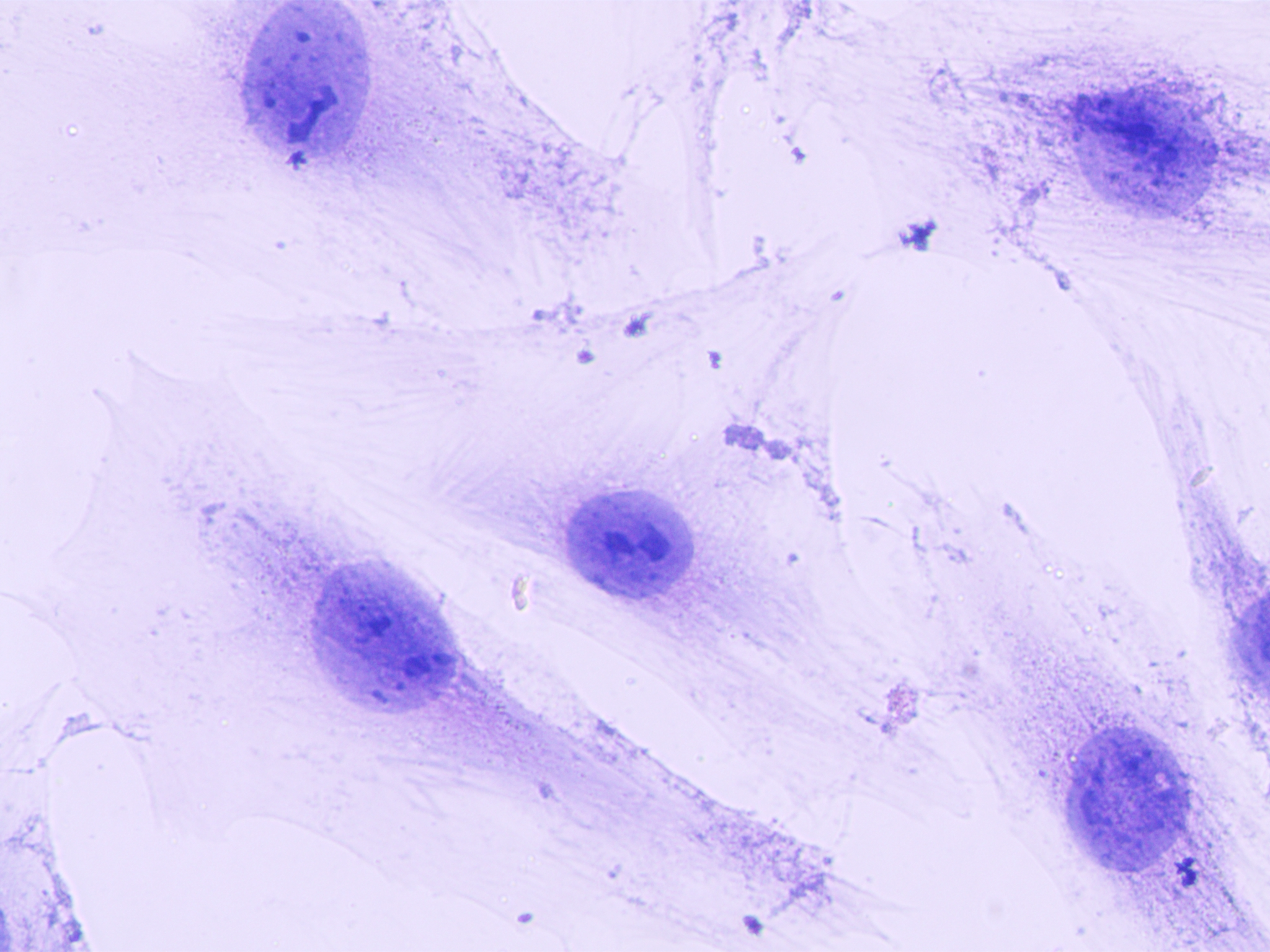

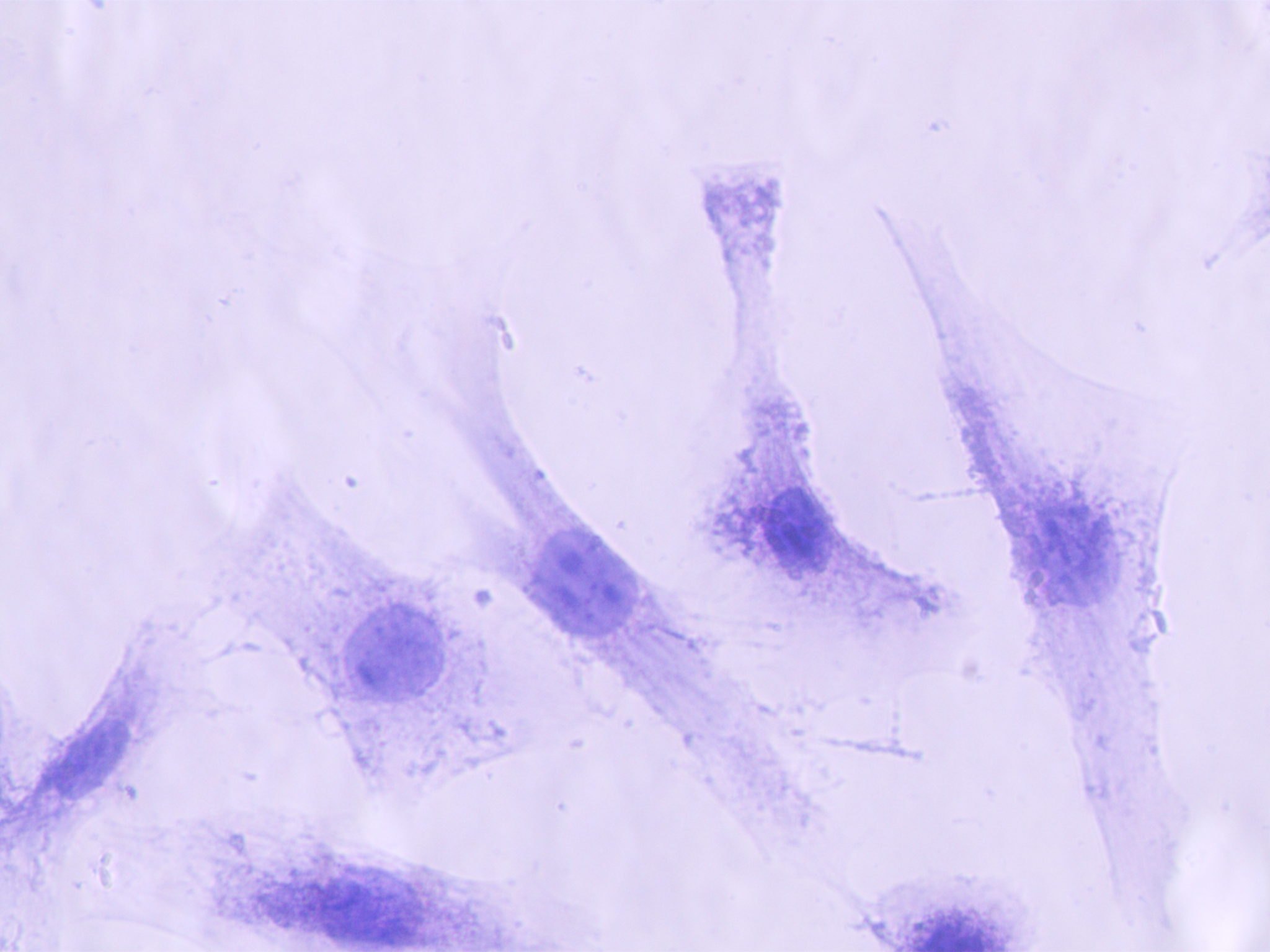


50 μm

**Figure S1**. Validation of morphological changes on senescent cells. Hematoxylin and eosin staining representing morphological alterations of senescent cells. Scale bars, 50 μm.


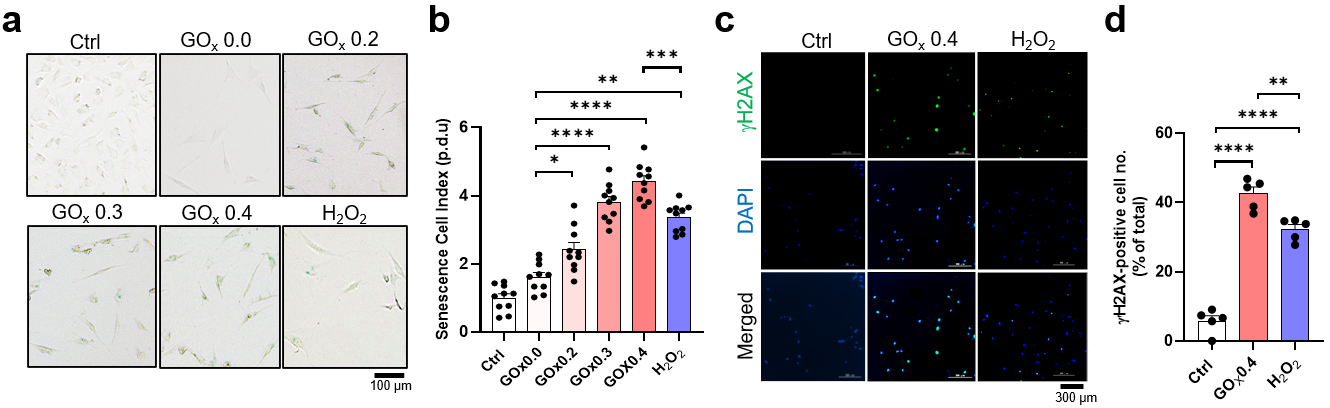


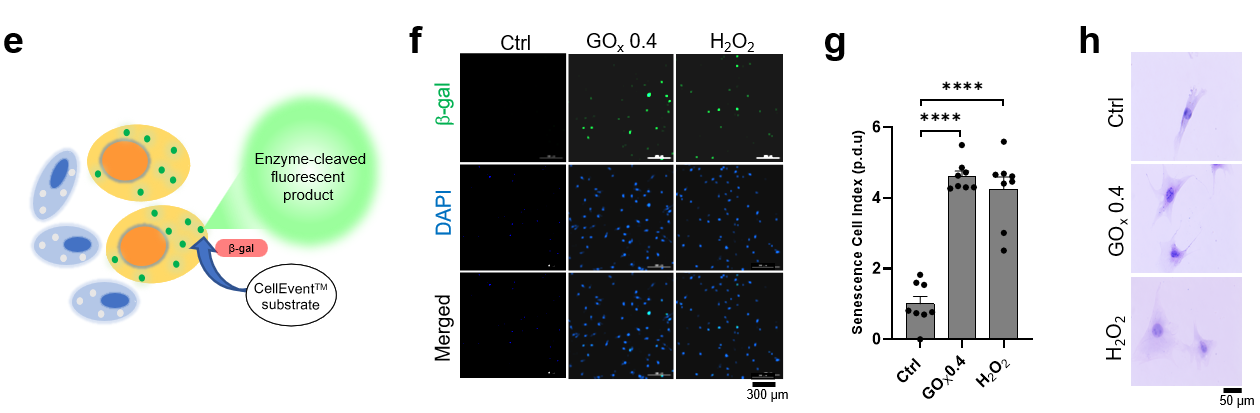


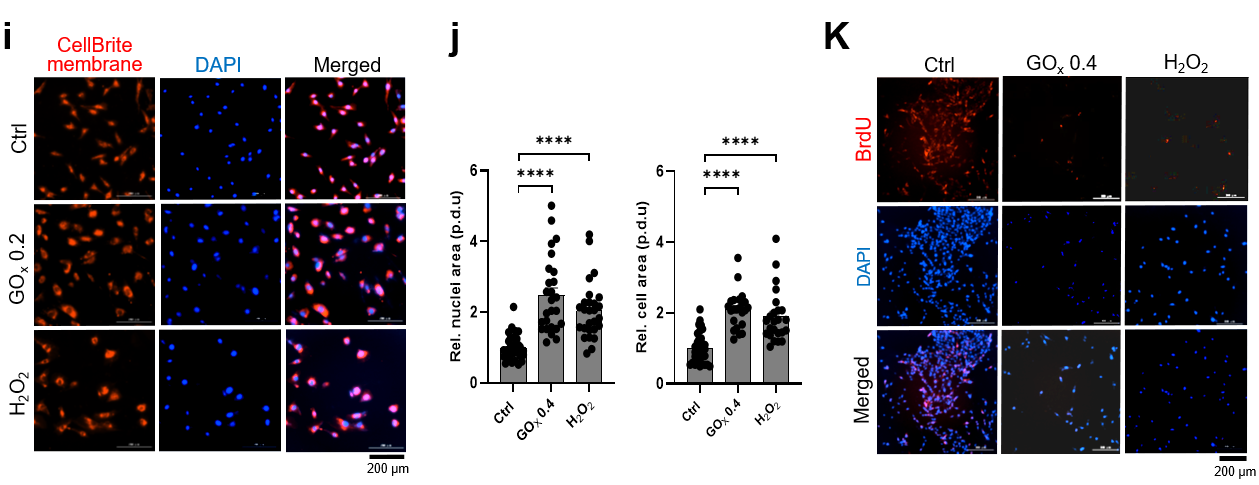


**Figure S2.** Validation of HRH-induced cellular senescence on p16 fibroblasts. a) Senescence induction effect was evaluated by SA-β-Gal staining with quantitative results (b). Scale bars, 100 μm. c-g) Further confirmation were conducted by γH2AX staining (c) with quantitative results (d), and CellEvent staining (f) with conceptual protocol (e) and quantitative results (g). Scale bars, 300 μm. h-j) Morphological alterations of senescent cells were evaluated by Hematoxylin and eosin staining (h) (Scale bars, 50 μm), and quantified by CellBrite membrane staining (i) with quantitative results (j) (Scale bars, 200 μm). K, BrdU incorporation assay indicating cell proliferation. Scale bars, 200 μm. The quantitative results are shown as mean ± SEM. Significant levels were set at *P< 0.05, **P < 0.01, ***P < 0.001, ****P < 0.0001.


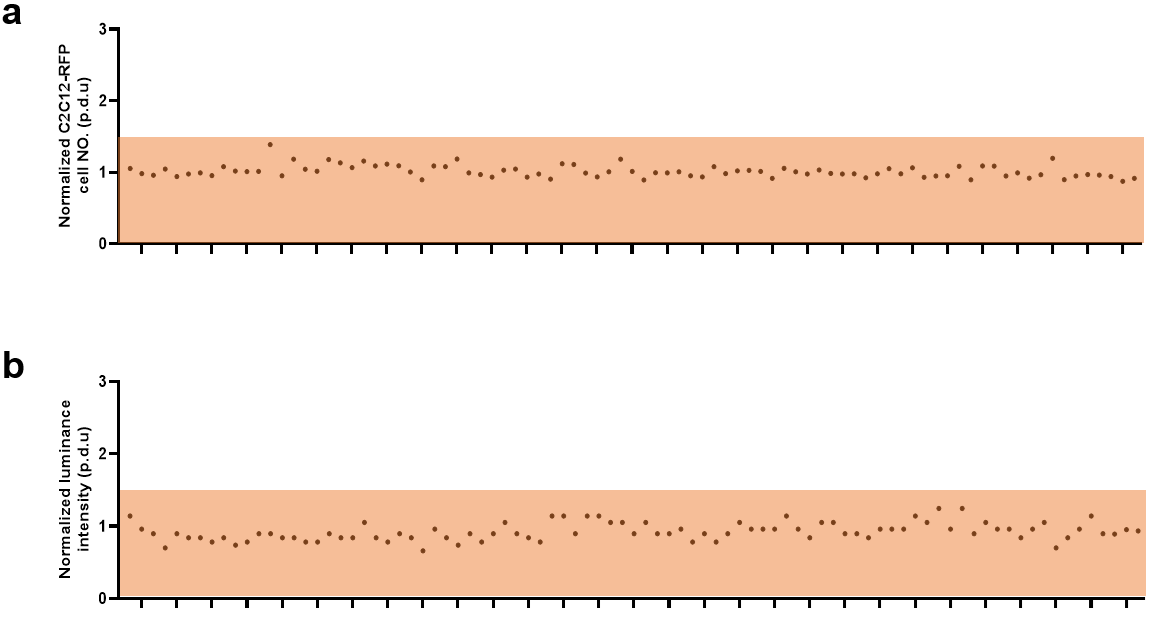


**Figure S3**. Toxicity testing for potential senolytics. a) Drug toxicity of 29 potential senolytics was measured by C2C12-RFP cell numbers after administration. Orange area representing cell numbers with no significant alterations, indicating drug safety. b) Drug toxicity of 29 potential senolytics was validated by luciferase intensity of normal p16-luc cells after administration. Orange area representing luminance with no significant alterations, indicating drug safety.
